# Development of AlN/Epoxy Composites with Enhanced Thermal Conductivity

**DOI:** 10.3390/ma10121442

**Published:** 2017-12-18

**Authors:** Yonggang Xu, Chi Yang, Jun Li, Xiaojian Mao, Hailong Zhang, Song Hu, Shiwei Wang

**Affiliations:** 1 Key Laboratory of Transparent Opto-Functional Inorganic Materials, Shanghai Institute of Ceramics, Chinese Academy of Sciences, Shanghai 201899, China; ygxu@student.sic.ac.cn (Y.X.); leejimmy@mail.sic.ac.cn (J.L.); seadragon@mail.sic.ac.cn (H.Z.); yzhusong@163.com (S.H.); 2 University of Chinese Academy of Sciences, Beijing 100049, China; 3 Beijing Institute of Space Long March Vehicle, Beijing 100076, China; yangchilxs@163.com; 4 The State Key Lab of High Performance Ceramics and Surperfine Microstructure, Shanghai Institute of Ceramics, Chinese Academy of Sciences, Shanghai 200050, China

**Keywords:** composites, thermal conductivity, AlN porous ceramics, infiltrating

## Abstract

AlN/epoxy composites with high thermal conductivity were successfully prepared by infiltrating epoxy into AlN porous ceramics which were fabricated by gelcasting of foaming method. The microstructure, mechanical, and thermal properties of the resulting composites were investigated. The compressive strengths of the AlN/epoxy composites were enhanced compared with the pure epoxy. The AlN/epoxy composites demonstrate much higher thermal conductivity, up to 19.0 W/(m·K), compared with those by the traditional particles filling method, because of continuous thermal channels formed by the walls and struts of AlN porous ceramics. This study demonstrates a potential route to manufacture epoxy-based composites with extremely high thermal conductivity.

## 1. Introduction

In recent years, polymer composites have been widely applied in the field of electronic devices, including central processing units, radio frequency units, batteries, and displays [1], for their outstanding dielectric properties, easy fabrication, excellent chemical resistance, and low cost [2,3,4]. With the miniaturization of microelectronics, it is necessary to fabricate new polymer composites with high thermal conductivity so that they can successfully be used in electronic devices [5,6], with an extended lifetime for the devices, especially in high operating temperatures [7]. However, traditional polymers such as polyethylene (PE) and polypropylene (PP) can hardly dissipate heat effectively due to their low thermal conductivity (~0.2 W/(m·K)) [8,9]. Therefore, it is highly desirable to increase the thermal conductivity of traditional polymers [10].

Normally, the most commonly used way to increase the thermal conductivity of polymer matrix composites includes dispersing highly thermally conductive fillers such as diamond, AlN, SiC, BN, or Si_3_N_4_ into polymer matrixes [3,11]. However, a high loading fraction of these fillers is generally needed to achieve a satisfactory thermal conductivity. For instance, to achieve a thermal conductivity of 2 W/(m·K), a loading fraction of more than 30 vol % is required for BN, AlN, and Si_3_N_4_ [11,12,13,14,15]. However, with the increasing solid content, the machinability of the composites degenerates [16]. Furthermore, the high loading fraction of filler is also detrimental to the mechanical performance of the polymer matrix composites [17,18], which greatly limits the application of these materials. For these reasons, it is of great importance to manufacture polymer matrix composites with high thermal conductivity at a low loading fraction. 

Virtually, former studies showed that the low thermal conductivity of polymer matrix composites at a low loading fraction was mainly ascribed to the fact that thermally conductive fillers were randomly distributed in the matrix, which did not form a thermal conductive path [10,19]. We proposed the construction of a thermal conductive path in epoxy matrix using AlN porous ceramic as the reinforcement, because AlN has high thermal conductivity.

In this work, 3D AlN porous ceramics with high thermal conductivity and excellent mechanical performance were prepared using a novel gelling system [20] and mechanical foaming [21]. Then, epoxy was infiltrated into these sintered 3D AlN porous ceramics to fabricate AlN/epoxy composite. It is demonstrated that the 3D AlN porous ceramics could greatly increase the thermal conductivity of epoxy at a low loading fraction.

## 2. Results and Discussion

### 2.1. Powder Treatment

**Figure 1:**
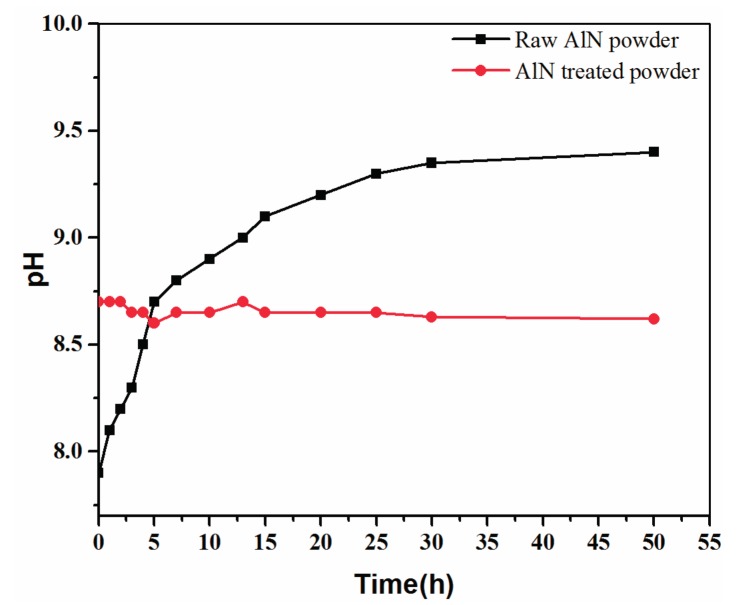
Variation of pH in aqueous AlN suspension containing treated and untreated AlN powder.

Aqueous slurry is generally required in order to fabricate AlN porous ceramics. However, it is known that AlN powder has high reactivity with water [22], forming alkaline solution [23,24]. Figure 1 illustrates the pH value variation of the aqueous suspension containing 1 wt % AlN powder, as well as that of the treated AlN powder suspension for comparison. It is clear that the pH value of the aqueous suspension with untreated AlN powder increases from 7.9 to 9.3 in 50 h, indicating the occurrence of the hydration reaction. The pH value of the suspension with treated AlN powder remains constant at 8.7 within the same period, which indicates the effectiveness of the water-resistance treatment. Additionally, the stable period over 50 h is sufficient for following foaming and gelation.

**Figure 2:**
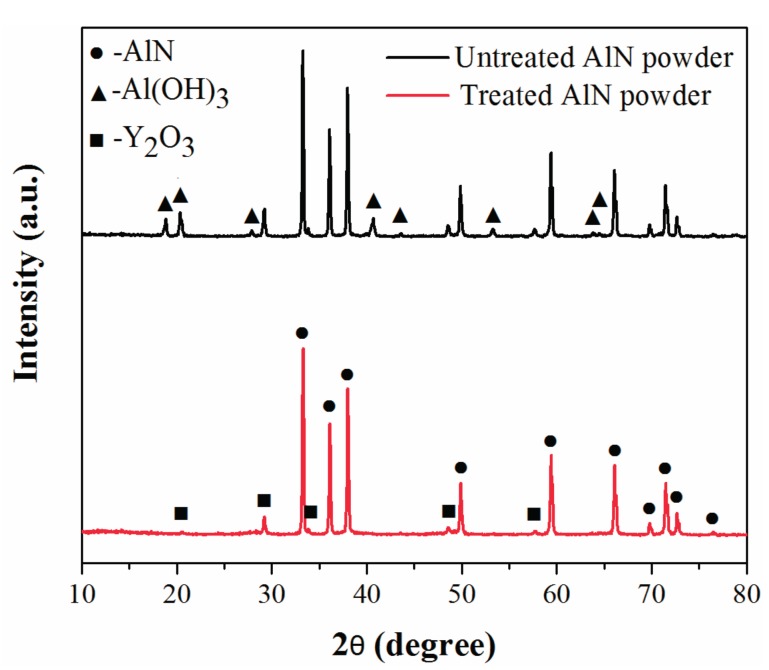
XRD patterns of the untreated and treated AlN powder dried from the suspensions.

The resulting products dried from the suspensions were examined by X-ray diffractometer (XRD). As shown in Figure 2, the hexagonal AlN is the dominating phase for the treated powder, with a small amount of Y_2_O_3_ which was added as the sintering aid. Meanwhile, in the sample of the untreated powder, the diffraction peaks of Al(OH)_3_ are evidently detected, indicating the reaction of untreated AlN powder with water [24]. Hence, the XRD data is additional valid evidence to confirm that the water-resistance treatment is effective to protect AlN against hydrolysis.

### 2.2. Properties of AlN Porous Ceramics

**Figure 3:**
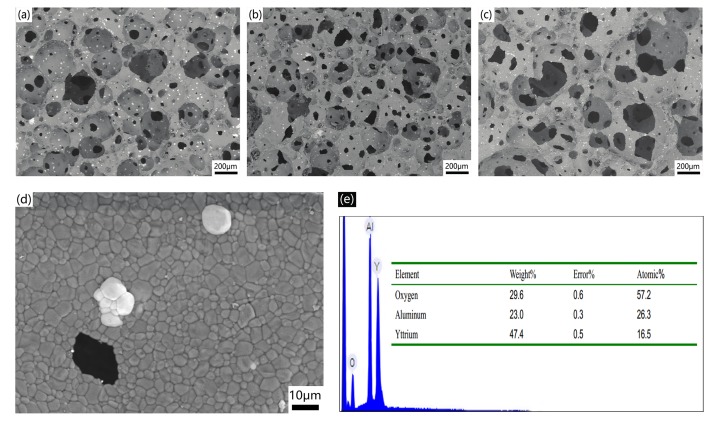
Microstructures of the AlN porous ceramics with different porosities (**a**) 72.1%; (**b**) 76.2%; (**c**) 81.3%; (**d**) morphology of the cell wall in the AlN porous ceramics with a porosity of 72.1%; (**e**) energy dispersive spectroscopy (EDS) spectrum of the white spot in (**d**).

The morphologies of the AlN porous ceramics manufactured using suspensions with solid loading of 48 vol %, 45 vol %, and 42 vol % are shown in Figure 3, with the corresponding porosities of 72.1%, 76.2%, and 81.3%. All AlN porous ceramics exhibited cellular structure with approximately spherical cells with no preferred orientation. It could also be observed that with the increase of porosity, the degree of the pore connection between spherical cells becomes remarkable, which is beneficial for further impregnation of epoxy solution to fabricate AlN/epoxy composites. Figure 3d shows the detailed structure of the cell wall of the AlN porous ceramics. The cell wall was constructed by numbers of AlN crystalline grains without obvious micropores. The average grain size of AlN in the cell wall was less than 10 μm. The grain boundaries were very clear, which is believed to be beneficial for improving the thermal conductivity [25]. It can also be seen that there were two white spots segregated on the surface of the cell wall. Figure 3e shows the energy dispersive spectroscopy (EDS) results of the white spot. Obviously, the white spot is composed of Al, O, Y elements. It is considered that the sintering aid Y_2_O_3_ reacts with Al_2_O_3_ during sintering to form Y, Al, and O ternary compounds [26], which is not soluble in the AlN crystal lattice.

**Figure 4:**
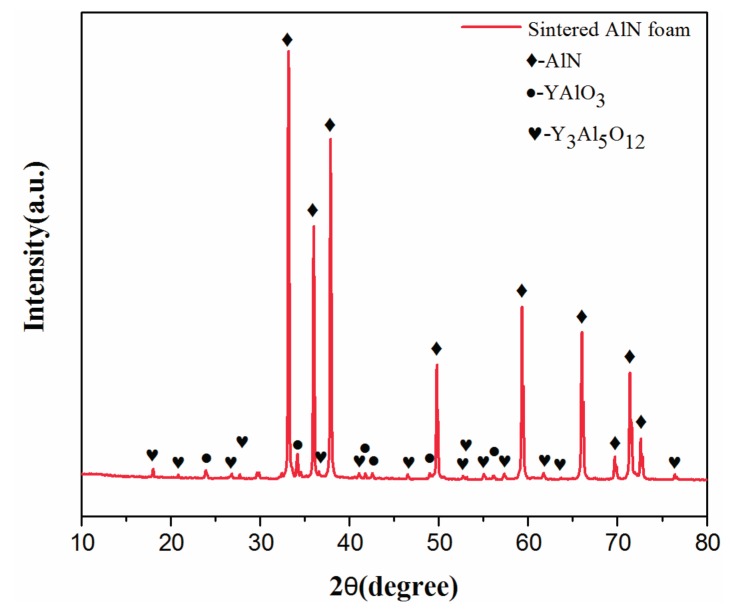
Powder XRD pattern of the AlN porous ceramic with the porosity of 72.1%.

The XRD pattern of the AlN porous ceramic is shown in Figure 4. The result shows that besides the dominant AlN phase there are small amounts of YAlO_3_ and Y_3_Al_5_O_12_. The component is in accordance with the result of EDS as shown in Figure 3e, which indicates the generation of yttrium aluminate by the reaction of Y_2_O_3_ and Al_2_O_3_ [27]. Hence, adding Y_2_O_3_ may not only accelerate the sintering procedure, but may also reduce the oxygen content in the AlN grains. It is notable that the precipitate phase distributes on the surface of the cell wall (as shown in Figure 3d), instead of in the grain boundaries, which is believed to be beneficial for improving the thermal conductivity of the AlN porous ceramics.

### 2.3. Properties of AlN/Epoxy Composite

**Figure 5:**
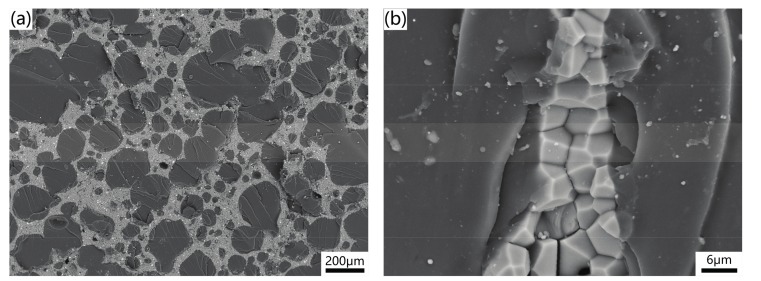
(**a**) Fracture surface of the composite reinforced by the AlN porous ceramic with 72.1% porosity; (**b**) interface between AlN and epoxy.

The AlN porous ceramics shown in Figure 3 were selected to fabricate AlN/epoxy composites by infiltrating epoxy resin into the porous cells. After curing, the AlN/epoxy composites were obtained. The cross-section of the resulting composite with 72.1% porosity is shown in Figure 5. The bright region represents the AlN skeleton with cellular structure, while the spherical black region corresponds to the infiltrated epoxy owing to the difference in the atomic mass. The structure of the AlN porous ceramic remained intact after infiltration, and most cells were filled with the epoxy. The interface of AlN and epoxy is shown in Figure 5b. It can be seen that the epoxy and AlN skeleton were tightly packed together, which would result in high mechanical strength and thermal conductivity.

**Figure 6:**
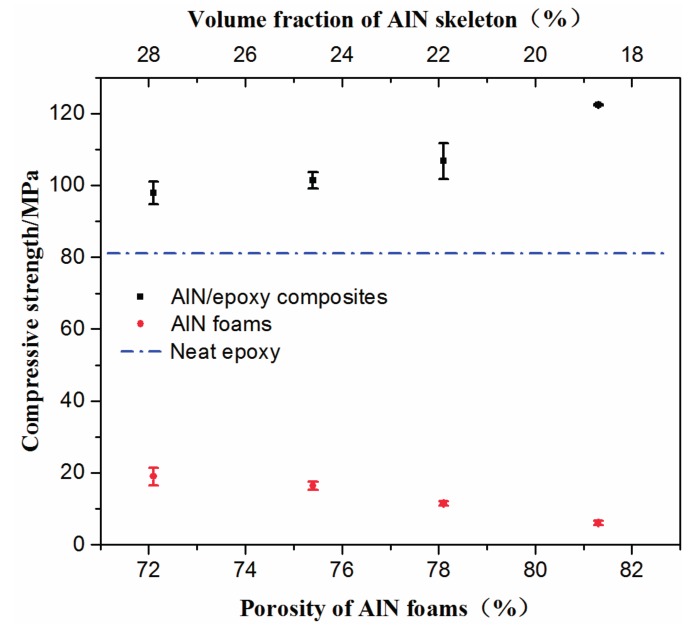
Compressive strength of AlN/epoxy composite and epoxy matrix, respectively.

Compressive strength of the composites containing different volume fraction of AlN skeleton, as well as the original AlN porous ceramics are shown in Figure 6. The compressive strength of AlN porous ceramics increased from 6 MPa to 19 MPa as the porosity decreased from 81.3% to 72.1%. Though the compressive strengths of the AlN porous ceramics were not high, they may survive during the infiltration and curing of the epoxy resin. As expected, the compressive strengths of the AlN/epoxy composites were much higher than those of the original AlN porous ceramics, and even higher than the pure epoxy (~82 MPa). However, it is obvious that the compressive strength of the composites decreased with the increase of the AlN fraction in the investigated range. This could be attributed to the difference of the structures of the AlN porous ceramics. In the composites, the epoxy matrix is divided into many spheres filled in the cells of the original AlN porous ceramic, as shown in Figure 5. When the fraction of AlN in the composite increased in the present case, the degree of the pore connection of the original AlN porous ceramics decreased, as shown in Figure 3a–c. Hence, these isolated epoxy spheres have less degree of connection to each other, which means that the epoxy network is easy to break. It could be confirmed by the original stress-strain curves that the stress declined to a platform after achieving the maximum value, which shows a combination of the curves for brittle ceramics [28] and plastic polymers [29,30].

**Figure 7:**
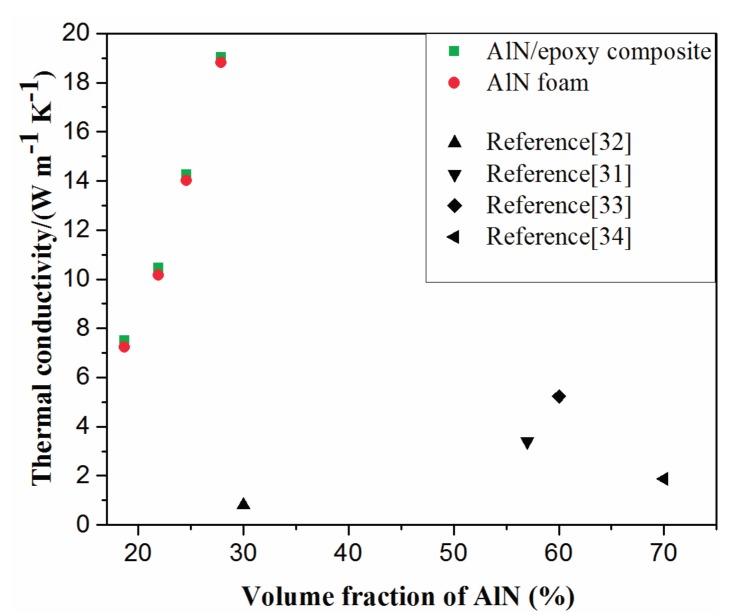
Thermal conductivities of the AlN foams, the AlN/polymer composites, and AlN/polymer composites reported elsewhere [31,32,33,34].

The thermal conductivities of the AlN/epoxy composites and the corresponding AlN porous ceramics are plotted in Figure 7. The thermal conductivity of the AlN/epoxy composites increased from 7.5 W/(m·K) to 19.0 W/(m·K) with the fraction of the AlN skeleton increasing from 19.7% to 27.9%, which are two orders of magnitude higher than that of the pure epoxy (0.2 W/(m·K)). It can also be easily found that the thermal conductivities of the composites were slightly higher than AlN porous ceramics, which means that the AlN skeleton was the dominant contribution to the thermal conductivity of the composite. The values of AlN filled epoxy composites reported in the literature are also shown in Figure 7 for comparison. It could be easily found that the thermal conductivities of the AlN/epoxy composites were dramatically advanced by using the manufactured AlN porous ceramics as skeleton instead of filling powders or whiskers directly. For example, for composite containing about 30 vol % AlN, the thermal conductivity of the present work was about 25 times higher than that using AlN powder as reported by Hu et al. [31]. The 3D network of AlN porous ceramic which has high thermal conductivity forms a channel for heat transport, while in the AlN particle dispersed composite, heat has to transport through polymer matrix as well as the interface, since the particles are not connected. Another advantage which is beneficial for high thermal conductivity is that the AlN porous ceramics have dense cell walls with clean grain boundaries by removing oxygen contamination from AlN grains, as shown in Figure 3 and Figure 4.

## 3. Experiment and Characterization

### 3.1. Experimental Procedure

Commercial AlN powder (d_50_ = 1.8 μm, Tokuyama Soda Co., Tokyo, Japan) was selected as the starting material and was mixed with 3 wt % Y_2_O_3_ powder (5 N purity, Jiangyin Jiahua Advanced Materials Resources Co., Ltd., Jiangyin, China) as sintering aid. The mixed powder was modified with a commercial polyurethane and tetraethylene pentamine in ethanol by ball milling for 2 h to prevent hydrolysis. The resulting suspension was dried at 60 °C for 36 h. Then, the modified AlN slurries with 42–48 vol % solid loading and 0.5 wt % Isobam 104 (a copolymer of isobutylene and maleic anhydride, Kuraray Co., Ltd., Osaka, Japan, simply noted as Isobam hereafter) were prepared by ball milling. Additionally, 1.0 wt % EMAL-TD (C_12_H_25_OSO_3_HN(C_2_H_4_OH)_3_) (Kao Chemical Co., Tokyo, Japan, noted as Surf-E hereafter) was added as foaming agent. A kitchen mixer was used to generate foams. After foaming, the slurries were cast into glass molds and gelled at room temperature (25 °C) for 4 h, then the green bodies were demolded and dried at room temperature (25 °C) for two days. Before sintering, the green bodies were calcined at 600 °C in air for 4 h with a heating rate of 1.0 °C/min to remove polyurethane, tetraethylene pentamine, and Isobam. Then, the green bodies were sintered at 1800 °C in nitrogen atmosphere for 6 h. Commercial epoxy resin (GCC-135) and its hardener (GCC-137) (Kunshan green follow chemical industry Co., Ltd., Kunshan, China) with mass ratio of 10:3 were used as the matrix in the present study. The mixed polymer was infiltrated into AlN porous ceramics by using a vacuum-assisted resin impregnation method. After the impregnation procedure, the AlN porous ceramics together with the mixed polymer solution were put into a drying oven at 60 °C for 1 h.

### 3.2. Characterization

The pH was tested by a digital pH meter (pHSJ-3F, Leici, Shanghai, China) at room temperature. Phase compositions of the samples were analyzed using an X-ray diffractometer (DX-2700, Haoyuan, Dandong, China) with Cu Kα radiation (λ = 0.15406 nm) with a step size of 0.03 degree and a scan speed of 0.6 s per step from 10 degree to 80 degree. Pore structure and fracture surface morphologies of the composites were observed by scanning electron microscopy (SEM, Hitachi TM3000, Hitachi, Lt., Tokyo, Japan), and the samples were coated by gold films as the conductive coatings. Energy spectrum analysis were carried out by energy dispersive spectroscopy (EDS) attached to the SEM instrument. The porosity of the AlN porous ceramics was tested using the Archimedes method. Compressive strength was measured using a universal testing machine (INSTRON-1195, Instron Co., Norwood, MA, USA) at a loading rate of 1 mm/min with a size of Φ 10 mm × 12 mm. Thermal conductivity was measured on cylinder samples (Φ 50 mm × 12 mm) using the guarded heat flow meter technique (DTC-300, TA Instruments Co., New Castle, DE, USA).

## 4. Conclusions

AlN/epoxy composites with high thermal conductivity have been successfully prepared by using AlN porous ceramics as the reinforcement. The AlN porous ceramics were fabricated by gelcasting of foaming method. The compressive strengths of the AlN/epoxy composites were enhanced compared with the pure epoxy by using these AlN porous ceramics as the reinforcement. The thermal conductivity of the AlN/polymer composites increased from 7.5 W/(m·K) to 19.0 W/(m·K) with the fraction of the AlN skeleton increasing from 19.7% to 27.9%, which are two orders of magnitude higher than that of the pure epoxy. For composites containing about 30 vol % AlN, the thermal conductivity of the present samples were about 25 times higher than those using the traditional particles filling method. This study demonstrates that using the 3D porous ceramics as the reinforcement is a potential route to manufacture epoxy matrix composites with extremely high thermal conductivity at a low loading fraction.
